# Supplementary material for: Deficits in Cerebellum-Dependent Learning and Cerebellar Morphology in Male and Female BTBR Autism Model Mice
Source: NeuroSci. 2022 Nov 9;3(4):624–44. doi: 10.3390/neurosci3040045 (PMC10292658; doi:10.3390/neurosci3040045)
Supplement: Supplementary file 1 [file neurosci-03-00045-s001.zip › neurosci-1944174-supplementary Figures.pdf]

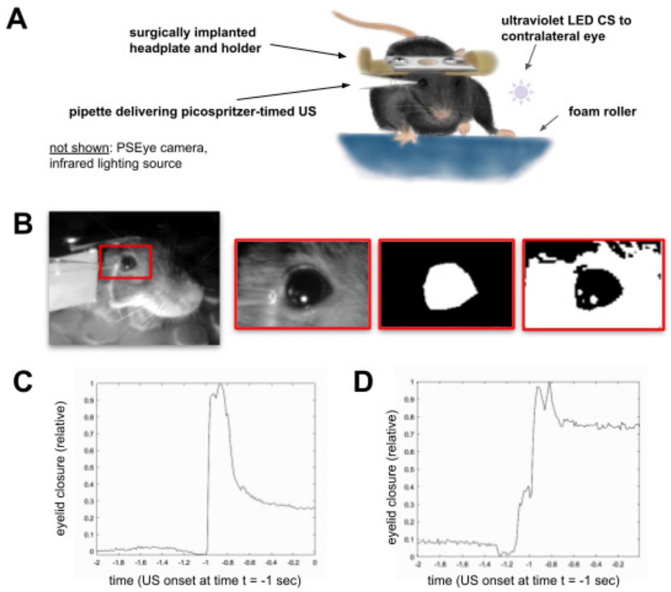

Supplementary Figure S1. Protocol for collecting and analyzing images from delay eyeblink conditioning sessions.

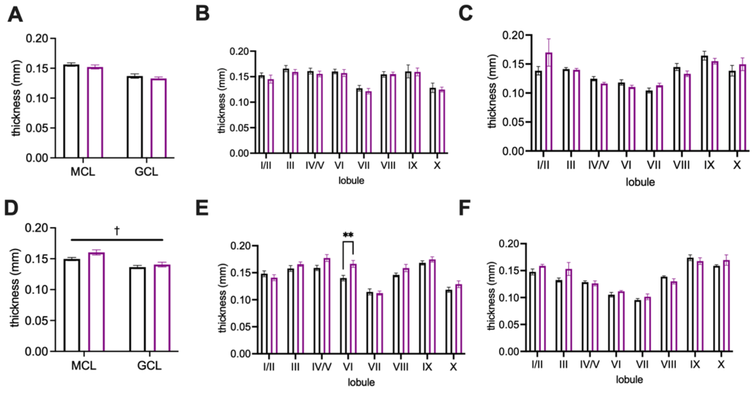

Supplementary Figure S2. The thicknesses of the molecular and granule cell layers depend on sex and lobule.

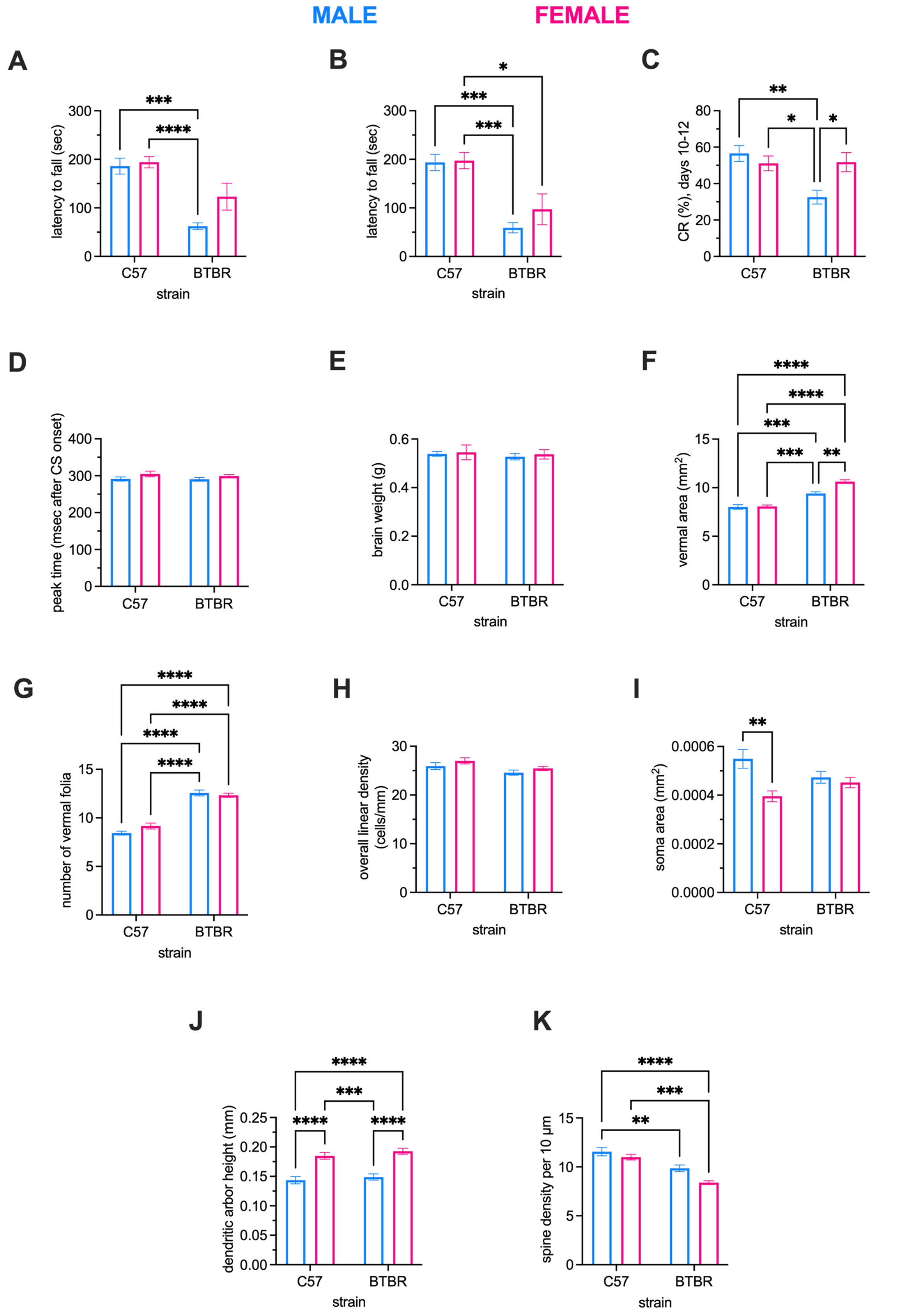

Supplementary Figure S3. Side-by-side sex and strain analysis of male and female experiments for BTBR and C57 mice. In each instance, blue indicates data from male mice and pink indicates data from female mice. (A,B) Rotarod results from training day 1 and training day 2 – related to Figure

1A-B. (C) Delay eyeblink conditioning performance – related to Figures 1D and 1G. (D) Delay eyeblink conditioning peak performance time – related to Figures 1E and 1H. (E) Brain weight – related to Figures 2A and 3A. (F) Vermal area – related to Figures 2C and 3C. (G) Vermal foliation count – related to Figures 2E and 3E. (H) Purkinje cell linear density – related to Figure 4. (I) Purkinje cell soma area – related to Figures 5C and 5G. (J) Purkinje cell dendritic arbor height – related to Figures 5D and 5H. (K) Purkinje cell dendritic spine density – related to Figures 5E and 5I. Error bars denote standard error of the mean. Asterisks denote significant results from post hoc comparisons following a significant two-way ANOVA. *, $p < 0.05$; **, $p < 0.01$; ***, $p < 0.001$; ****, $p < 0.0001$. *, $p < 0.05$; **, $p < 0.01$; ***, $p < 0.001$; ****, $p < 0.0001$.
